# Experiences of Suicidality Following Discharge From a Mental Health Inpatient Unit: A Systematic Review and Meta‐Synthesis

**DOI:** 10.1002/cpp.70234

**Published:** 2026-02-11

**Authors:** Connor Heapy, Gillian Haddock, Jordan Parkinson, Daniel Pratt

**Affiliations:** ^1^ Division of Psychology and Mental Health, School of Health Sciences and Manchester Academic Health Science Centre University of Manchester Manchester UK; ^2^ Greater Manchester Mental Health NHS Foundation Trust Suicide, Risk and Safety Research Unit and the Manchester Academic Health Science Centre Manchester UK

**Keywords:** discharge, mental health inpatient unit, meta‐synthesis, psychiatric ward inpatient, review, suicide

## Abstract

People are at increased risk of suicide following discharge from inpatient mental health units. Understanding the reasons for this increased risk is important for reducing the number of people who die by suicide. Whilst reviews of quantitative research have identified risk factors, no reviews of the qualitative literature exist which could provide more nuanced explanations of elevated suicide risk during the post‐discharge period. This systematic review is the first to meta‐synthesise qualitative research on experiences of suicidality after being discharged from inpatient mental health units. We searched PsycINFO, MEDLINE, Web of Science, PubMed and ProQuest using relevant search terms. We identified 29 studies that met inclusion criteria and were included in the review. We analysed the data using thematic synthesis and identified five analytic themes: (1) Feeling prepared for the transition home, (2) Returning from safety to everyday hardship, (3) The need for connection and understanding, (4) Feeling neglected by the system, (5) Taking the reins on recovery. This review indicates that reducing post‐inpatient discharge suicides could be achieved through collaborative discharge preparation, immediate and intensive post‐discharge support, and empowering service‐user recovery.

## Summary

- Studies of experiences of suicidality following discharge from a mental health unit were reviewed.
- Poor experiences of discharge and aftercare were described by participants as increasing suicide risk.
- Relationships and being treated with compassion were described by participants as being protective against suicide risk.
- Clinicians should work to ensure discharge from mental health units is predictable, supportive, personalised, and empowering.

## 1 Introduction

Around 700,000 people are recorded as dying by suicide each year, making it the 15th leading cause of death worldwide (Dattani et al. 2023a; World Health Organisation, WHO 2021). These figures are likely underestimates since suicide is stigmatised around the world and illegal in some countries, meaning deaths by suicide may have been inaccurately categorised (Dattani et al. 2023b; Mishara and Weisstub 2016). The negative impact of suicide is much broader than the number of deaths suggests, with 20 suicide attempts for every suicide death (WHO 2021), and approximately 135 people being exposed to (i.e., reported knowing the person) every suicide death (Cerel et al. 2019). In addition, suicidal ideation can be highly distressing and is a key risk factor for suicide attempts and deaths (Rossom et al. 2017). Approximately 9% of the general population will experience suicidal ideation during their lifetime (Nock et al. 2008). Suicide attempts and deaths also pose a significant financial cost for the world economy (e.g., Kinchin and Doran 2017; Shepard et al. 2016).

One group of people at high risk of suicide are inpatients of mental health services, or those who have recently been discharged from such wards (Chung et al. 2017). Though there is some variation in design and function worldwide, such inpatient mental health services tend to offer 24‐h care for people with significant mental health difficulties who are deemed a risk to themselves or others (Johnson et al. 2022; Saya et al. 2019). These wards tend to offer assessment, management and intervention of severe mental health difficulties and associated risk, and patients may be on the wards voluntarily or involuntarily (Johnson et al. 2022; Saya et al. 2019). Despite 24‐h inpatient care, deaths by suicide in these settings still occur at a higher rate than in the community, but are highly variable. For example, in a meta‐analysis, Walsh et al. (2015) found a pooled estimate of around one death per 676 admissions worldwide, but individual studies included in the meta‐analysis reported rates of between one death per 171 admissions and one death per 3032 admissions. For the post‐discharge period, a systematic review and meta‐analysis of international studies found the rate of suicide deaths during the first 3 months post‐discharge from such wards was 100 times higher than for the global general population, with no differences in suicide deaths between continents (Chung et al. 2017). Another meta‐analysis of international studies found that the first week and month following discharge are particularly high‐risk periods for suicide (Chung et al. 2019), with 2950 suicides per 100,000 person years and 2060 suicides per 100,000 person years, respectively. Similarly, a study using all UK suicide data, between the years of 2011 and 2021, found that one fifth of all mental health patient suicide deaths were either by inpatients or those recently discharged from an inpatient ward (National Confidential Inquiry into Suicide and Safety in Mental Health [NCISH] 2024), with the highest number of suicides occurring in the first 2 weeks after discharge. Together, these findings suggest the period immediately following discharge confers the highest risk of suicide, with this risk slowly declining over time.

Whilst rates of inpatient and post‐discharge suicide deaths have been falling in some countries (e.g., Taiwan; Tseng et al. 2019), they are increasing or staying stable in others (e.g., Denmark; Madsen et al. 2020; United Kingdom; NCISH 2023). Several reviews have investigated a range of discharge interventions in an attempt to reduce suicide deaths—such as telephone contact, postcards, home visits and community outreach programmes—but have not identified consistent evidence that any of these interventions are effective in reducing suicides (Chaudhary et al. 2020; Tay and Li 2022; Tyler et al. 2019). Outside of the inpatient setting, literature reviews have shown that psychological interventions are effective in reducing suicidal ideation and attempts across diverse mental health populations, such as people diagnosed with depression, personality disorders and post‐traumatic stress disorder (Meerwijk et al. 2016; Méndez‐Bustos et al. 2019; van Ballegooijen et al. 2025). However, effect sizes are small–medium for the reduction of suicidal ideation and attempts, and many people do not respond to these interventions, so it remains unclear which are the key mechanisms offering patient benefit. In addition, there is a dearth of studies looking into the effectiveness of psychological interventions on suicidal ideation and attempts specifically adapted to the needs of high‐risk groups, such as mental health inpatients or those recently discharged from mental health wards. One exception is the Inpatient Suicide Intervention and Therapy Evaluation (INSITE) trial which demonstrated that a cognitive‐behavioural intervention designed to reduce suicidal ideation and behaviours (the Cognitive Behavioural Suicide Prevention [CBSP]; Tarrier et al. 2013) could be implemented successfully and safely in an inpatient setting (Haddock et al. 2019). However, this was a pilot trial and therefore efficacy could not be established. In summary, there is a need for further research to inform intervention development that reduces suicide ideation and behaviour, including deaths, amongst those experiencing the most acute risk.

Understanding the reasons why this inpatient and post‐discharge period is particularly risky for patients is crucial for informing future interventions. Both quantitative and qualitative studies have explored this question. Three systematic reviews, two of which involved meta‐analyses, have investigated the risk factors associated with post‐discharge suicide (Large et al. 2011; O'Connell et al. 2021; Tai et al. 2025). Risk factors that appear most significantly and consistently across these reviews include being male, having a history of self‐harm or suicide attempts, experiencing suicidal thoughts, experiencing depression, experiencing hopelessness and adverse life events. Protective factors include participation in aftercare and being aged under 30 or over 65 years old. However, effect sizes associated with these risk factors were modest and study heterogeneity was high. Together, these findings are not particularly helpful for intervention development as several of the identified risk factors are not modifiable in the inpatient or post‐discharge period. In addition, many of the identified risk factors are general risk factors for suicide (Franklin et al. 2017), and the findings lack a nuanced explanation about why the inpatient and/or post‐discharge period is particularly risky for service‐users. Indeed, the most recent of these systematic reviews (Tai et al. 2025) did not find the strongest suicide risk factors declined over time, leaving the declining rates of suicide in the post‐discharge period unexplained. The authors of this review suggested the need for research into important aspects of patient discharge, such as loss of hospital support, stigma and loss of social role associated with hospitalisation.

Qualitative research into the post‐discharge period can provide more nuanced explanations about suicide risks than quantitative research by highlighting the lived experiences of previous inpatients. Many qualitative studies have been conducted with current or recently discharged inpatients from a range of countries and have asked participants about their experiences of suicidality (i.e., suicidal thoughts and/or behaviours), support received and support needed during the discharge period (e.g., Awenat et al. 2018; Cutcliffe et al. 2012a; Owen‐Smith et al. 2014; Redding et al. 2017). Of course, there are limitations of qualitative research too, as participants may not be able to identify specific experiences that led to suicide. Rather, they may be able to describe a range of experiences that made the discharge period particularly difficult and therefore theoretically may have contributed to suicidality. However, understanding the issues that may contribute to suicidal thoughts or attempts in this period, either directly or indirectly, is crucial for reducing deaths. One important factor highlighted by interview studies (and in line with the previously described quantitative review by O'Connell et al. 2021), is the importance that patients place on being involved in the discharge process (e.g., Owen‐Smith et al. 2014). Another theme that helps explain why the post‐discharge period involves increased suicide risk is difficulties participants face around adapting to community life after spending significant periods of time in hospital (e.g., Redding et al. 2017). Despite many qualitative studies being conducted on this topic, a systematic review of this literature does not currently exist. Such a review would provide a collated and comprehensive understanding of people's experiences of this pre‐ and post‐discharge period and would complement the available quantitative literature.

The current study therefore aims to systematically review and meta‐synthesise the available qualitative literature on the suicide‐related experiences of individuals during the period of discharge from a mental health inpatient ward. Specific research questions explored included:

1. Why do participants think the discharge period is a time of increased suicidality?
2. What support do adults experiencing suicidality (i.e., suicidal thoughts/behaviours) believe they can, and cannot, access?
3. What support do adults experiencing suicidality believe they need to reduce the likelihood of suicide?

## 2 Methods

The protocol for this review was prospectively registered on the CRD PROSPERO website (Ref: CRD42024507691). The review methods were guided by the Preferred Reporting Items for Systematic Reviews and Meta‐Analyses (PRISMA; Page et al. 2021) and Enhancing Transparency in Reporting the Synthesis of Qualitative Research (ENTREQ; Tong et al. 2012) guidelines.

### 2.1 The Researchers

The research team was made up of individuals with varying personal and professional characteristics and experiences, meaning a range of perspectives were involved in this review. The analysis was conducted by the first author who is a clinical psychologist and researcher who has experience working on inpatient mental health wards and working with people experiencing suicidal thoughts or who have previously attempted suicide. All results were discussed with the other three authors. DP and GH are also clinical psychologists and experienced suicide researchers. JP has experience conducting suicide‐related research in inpatient mental health wards and working with people with mental health difficulties. The idea for the review was deemed important by a Patient and Public Involvement (PPI) group of people who had experienced suicidal thoughts or attempts and/or inpatient admission.

### 2.2 Search Strategy

A literature search was conducted in February 2024 and then updated in March 2025, across the databases of PsycINFO, MEDLINE, Web of Science and PubMed. ProQuest was also searched for grey literature. Based on PPI input, various third sector UK organisations (Samaritans, The McPin Foundation, National Survivor User Network, Survivor Research Network) were contacted for relevant research. No lower or upper date limits were used for the search. Key search terms included variations of ‘adult/service user’ and ‘suicide’ and ‘discharge/psychiatric ward’ and ‘qualitative/experience’. See Appendix A for full details of the search terms used.

Citation chaining was used to find papers missing from the initial search. That is, reference lists of included articles were searched for other articles that met inclusion criteria and included articles were searched using the ‘cited by’ function in Google Scholar.

### 2.3 Eligibility Criteria

Studies were included if the following criteria were met:

- Published in English and presented original data
- Published in peer‐reviewed journal, OR dissertation/thesis, OR published in a relevant UK third sector organisation's report
- Adult participants (aged ≥ 18 years).
- Used qualitative methodology (e.g., interviews, focus groups) and presented qualitative findings derived from an established qualitative analysis approach (e.g., thematic analysis). Where this criterion was met within a mixed‐methods study, the qualitative component was included, if reported distinctly.
- Participants were currently or had been an inpatient on a mental health ward for any period.
- Presented service‐users' first‐hand accounts of their experiences following discharge from a mental health inpatient ward OR presented first‐hand accounts of their thoughts about a future discharge. Such accounts may have included: why they felt suicidal; what support they received/felt they needed/will need.
- Indication that at least some participants experienced suicidal thoughts/feelings/urges, or demonstrated suicidal behaviour either before, during, or after discharge (e.g., suicidality stated in the inclusion criteria, indicated in a clinical variables table, or discussed in the results section).

Studies were excluded if any of the following criteria were met:

- Studies focusing only on non‐suicidal self‐harm
- Study/trial protocols
- Opinion/lobbying articles
- Only presented data from staff/carers
- Quantitative research, conference abstracts, case reports, and case series.
- Book chapters, editorials, reviews.
- Only partially follows established qualitative analysis approach (e.g., only codes data with no reference to developing themes).
- Patients admitted to and discharged from a non‐mental health ward, such as an acute physical health ward.

### 2.4 Data Extraction and Synthesis

All studies found in the searches were downloaded into reference management software, Zotero (Version 6.0.21). Duplicates were automatically detected and manually removed. Remaining studies were then transferred to the Rayyan web app for systematic reviews (Ouzzani et al. 2016). Next, the first and third authors screened all titles and abstracts against eligibility criteria. The two reviewers had an agreement rate of 92%. When only one author chose to include an article, the full text article was reviewed to assess eligibility for inclusion. The first author then screened remaining studies at full‐text level against eligibility criteria and the third author screened 20% of randomly selected results. The two reviewers had an agreement rate of 90%. Disagreements were resolved through discussion between the first and third authors. If agreement could not be reached, further disagreements were resolved through discussion with the second and fourth authors. At both stages, each reviewer was blind to the decision of the other.

After screening, the following data were extracted from remaining studies into a Microsoft Excel spreadsheet: author, year, country, method of data collection (e.g., interview, focus group), method of data analysis (e.g., thematic analysis, IPA), sample size, sample characteristics (e.g., age, gender, mental health diagnoses). The results section of each paper was copied into NVivo software. The data were then synthesised thematically using an inductive approach to identify common themes across studies following three stages outlined by Thomas and Harden (2008): (1) line‐by‐line coding of the results section of each study, (2) developing descriptive themes from the initial codes, (3) developing analytical themes from the descriptive themes. Descriptive themes and analytical themes were developed by the lead researcher (CH) and then discussed with co‐authors. Thematic synthesis was chosen as a flexible and iterative approach which allows overarching themes to be drawn from studies with diverse qualitative designs.

### 2.5 Quality Assessment

The Critical Appraisal Skill Programme (CASP 2018) checklist was used by the first author to assess quality and risk of all included studies. Next, the third author quality assessed 45% (*n* = 13) of included papers. The two reviewers had an agreement rate of 84%. Disagreements were resolved through discussion between the first and third author. If agreement could not be reached, further disagreements were resolved through discussion with the second and fourth authors.

The CASP includes 10 questions covering the aims (e.g., ‘Was there a clear statement of the aims of the research?’), method (e.g., ‘Was the research design appropriate to address the aims of the research?’), results (e.g., ‘Is there a clear statement of findings?’) and discussion (e.g., ‘How valuable is the research?’, interpreted as ‘Is this research valuable’ so that this item can be scored on the same scale as the other items) of each study. Each question is answered either ‘Yes’, ‘No’ or ‘Cannot tell’ (a scoring system is not recommended by the CASP authors). For feasibility or mixed‐method studies, only the qualitative sections of the paper were quality assessed.

## 3 Results

### 3.1 Study Selection

**FIGURE 1:**
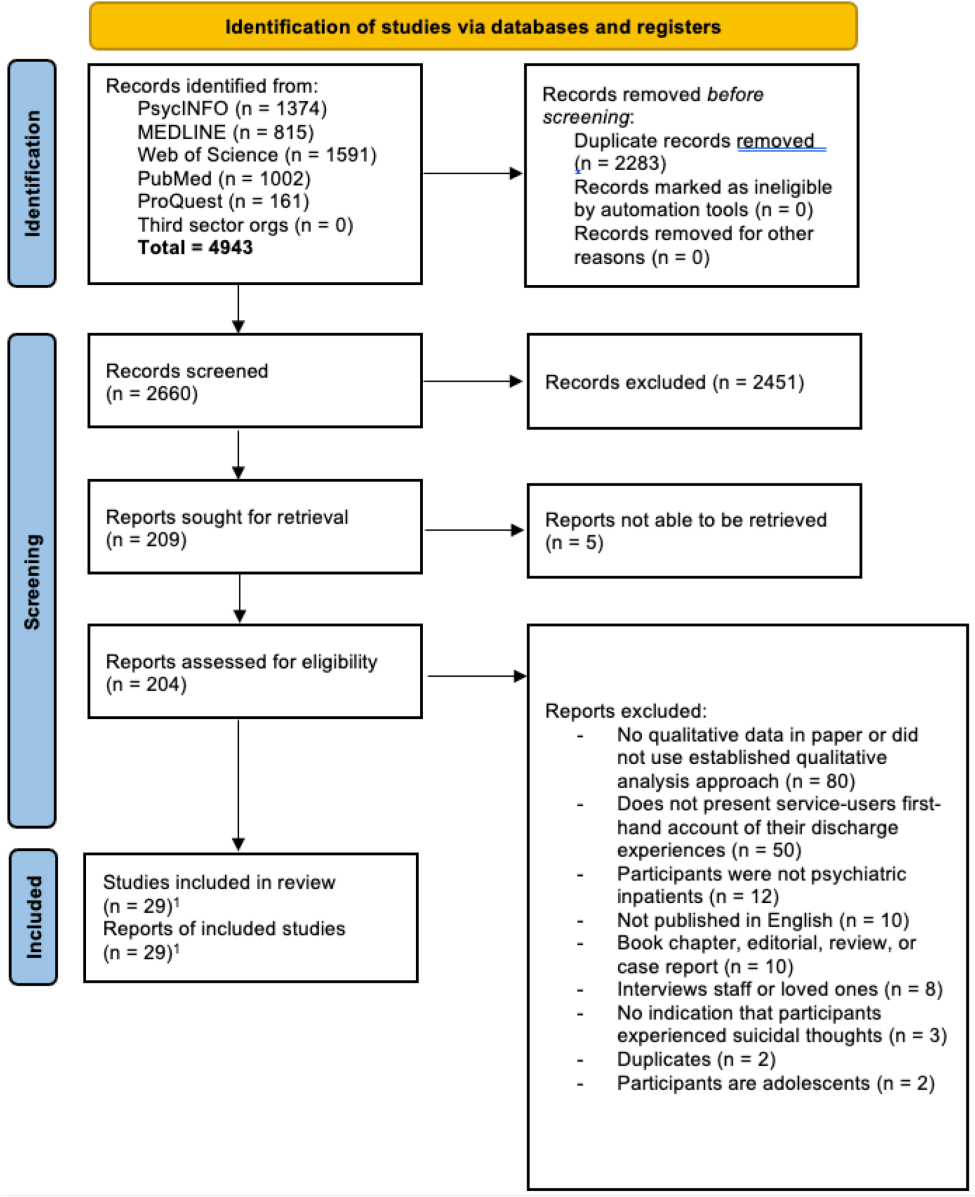
PRISMA diagram of study search and selection procedure. ^1^This figure includes two papers identified through citation chaining.

The results of the literature search are presented in the PRISMA diagram in Figure 1. In addition to studies found in the initial search, two studies were found from citation chaining. In total this provided 29 articles following the removal of duplicates, titles and abstracts, and full‐text articles.

### 3.2 Study Characteristics

Study characteristics are presented in Table 1. The included studies covered 11 different countries, with most studies coming from the United Kingdom/England and Wales (*k =* 9). Over 400 participants were included overall and the ages of included participants ranged from 18 to 67 years. Most data were collected by interview (*k* = 24).

**TABLE 1:** Methodological details of included studies.

Authors, year	Research question/aims	Country	Data collection method	Data analysis method	Sample size	Sample age range	Sample gender	Suicidal ideation/behaviours	Discharge status
Awenat et al. (2018)	To investigate suicidal in‐patients' views and expectations of a novel ward‐based suicide‐focussed psychological therapy intervention nested within a pilot feasibility clinical trial	United Kingdom	Interviews	Thematic analysis	20	22–65	14 males; 6 females	Recent suicidal ideation or behaviour (all participants)	Inpatients
Bahlmann et al. (2022)	To assess the feasibility and the acceptance of a suicide prevention intervention for inpatients following a suicide attempt.	Germany	Open‐ended questionnaire	Content analysis	20	n/a	11 males; 9 females	Recent suicide behaviour (all participants)	Inpatients
Bennewith et al. (2014)	To assess the usefulness and feasibility of a contact‐based intervention for people recently discharged from inpatient psychiatric care in the United Kingdom	United Kingdom	Interviews	Thematic analysis	13	n/a	8 males; 5 females	Indication of suicidality in results section	Discharged
Berg et al. (2020)	How do suicidal patients experience safe clinical practice during hospitalisation in mental health wards?	Norway	Interviews	Content analysis	18	18–57	7 males; 11 females	Recent suicidal ideation or behaviour (all participants)	Inpatients
Brenisin et al. (2023)	To investigate peer support workers lived experience of adjusting to independent community living following discharge from an inpatient ward.	United Kingdom	Interviews	Interpretative phenomenological analysis	4	n/a	1 male; 3 females	Indication of suicidality in results section	Three participants discharged (one participant never inpatient)
Chen et al. (2022)	To explore barriers and needs to increasing social connectedness following psychiatric hospitalisation	United States	Interviews	Grounded theory	30	n/a	26 male; 3 female; 1 transgender	Recent suicidal ideation (16/30 participants)	Some participants inpatients; some discharged
Coffey et al. (2019)	To identify factors that facilitate or hinder recovery‐focused personalised care planning and coordination in acute inpatient mental health settings	England and Wales	Interviews	Framework method	36	n/a	n/a	Indication of suicidality in results section	Inpatient
Cutcliffe et al. (2012a)	What is the nature of the experience of being discharged after being at risk for suicide as an essentially human, lived experience? How does it contribute to a person's ongoing risk of suicide?	Canada	Interviews	Methodological interpretation of phenomenology	20	n/a	10 males; 10 females	Recent or lifetime history of suicidal ideation or behaviour (all participants)	Discharged
Cutcliffe et al. (2012b)	What is the nature of the experience of being discharged after being at risk for suicide as an essentially human, lived experience? How does it contribute to a person's ongoing risk of suicide?	Canada	Interviews	Methodological interpretation of phenomenology	20	n/a	10 males; 10 females	Recent or lifetime history of suicidal ideation or behaviour (all participants)	Discharged
Fredriksen et al. (2020)	To investigate how individuals with depressive psychosis experienced inpatient treatment to affect suicidal thoughts and behaviour during an acute admission.	Norway	Interviews	Systematic text condensation	9	19–55	4 males; 5 females	Recent suicidal ideation or behaviour (all participants)	Inpatients
Fu et al. (2024)	To explore problems faced by patients after discharge, and their needs.	China	Interviews	Thematic analysis	17	n/a	10 males; 7 females	Indication of suicidality in results section	Discharged
Ghio et al. (2011)	To gain insight into the individual experiences of psychiatric patients who attempt suicide, to better understand causal and protective factors.	Italy	Focus groups	Thematic analysis	17	n/a	7 males; 10 females	Recent suicidal behaviour (all participants)	Inpatients
Hagen et al. (2018)	How did (former) suicidal inpatients experience treatment and care in psychiatric wards following the implementation of the National guidelines for prevention of suicide in mental health care? What can be improved in the treatment and care of suicidal inpatients?	Norway	Interviews	Interpretive phenomological analysis	5	33–54	1 male; 4 females	Recent suicidal ideation or behaviour (all participants)	Discharged
Hagen et al. (2020)	How do patients experience their suicidality and position as patients when they are hospitalised in a psychiatric acute ward, and how do they experience meetings with the professionals and the care they receive?	Norway	Interviews and participatory observation	Systematic text condensation	11	20–41	0 males; 11 females	Recent suicidal ideation or behaviour (all participants)	Inpatients
Hancock et al. (2022)	To understand the experiences of a peer support worker intervention on service users in the transition from inpatient ward to home.	Australia	Interviews and open‐ended questionnaire	Constant comparative analysis	17 interview; 12 completed questionnaire	n/a	Interview—9 males; 7 females; 1 other. Questionnaire – 4 males; 4 females; 4 not stated	Indication of suicidality in results section	Discharged
Heron et al. (2012)	To explore women's experiences of the process of recovery from postpartum psychosis and their beliefs about the services needed to support recovery	United Kingdom	Interviews	Grounded analytic induction approach	5	n/a	0 males; 5 females	Indication of suicidality in results section	Discharged
Jackson et al. (2020)	What were patients experiences of re‐engaging with life after their suicide attempt?	Australia	Interviews	Descriptive phenomenology	8	18–64	4 males; 4 females	Suicidal behaviour two or more years previous (all participants)	Discharged
O'Connor et al. (2023)	To determine the long‐term effects of a suicide prevention‐focused group therapy for veterans recently discharged from an inpatient psychiatry setting following a suicidal crisis.	United States	Interviews	Thematic analysis	30	n/a	n/a	Recent suicidal ideation or behaviour (all participants)	Discharged
Owen‐Smith et al. (2014)	To investigate the lived experience of discharge from psychiatric hospital and to explore the experiences of service users in the postdischarge period.	United Kingdom	Interviews	Thematic analysis	10	n/a	2 males; 8 females	Indication of suicidality in results section	Discharged
Pelto‐Piri et al. (2019)	To enhance our understanding of feelings of being safe or unsafe in psychiatric inpatient care from a patient perspective in the ward environment.	Sweden	Interviews	Content analysis	17	20–67	12 males; 5 females	Indication of suicidality in results section	Inpatients
Redding et al. (2017)	This study aims to explore what it is like for people being discharged from a psychiatric hospital stay.	United Kingdom	Interviews	Interpretative phenomenological approach	8	26–65	3 males; 5 females	Indication of suicidality in results section	Discharged
Samuelsson et al. (2000)	To describe the attempted suicide patient's perceptions of receiving specialised in‐patient psychiatric care.	Sweden	Interviews	Content analysis	18	18–53	12 males; 6 females	Recent suicidal behaviour (all participants)	Inpatients
Steinberg et al. (2024)	To improve and adapt an existing intervention to support care transitions and sustain patient wellbeing following discharge from an inpatient psychiatric unit.	Canada	Focus group	Grounded constructionist theory approach	15	n/a	2 males; 10 females; 1 non‐binary^a^	Experienced suicidal ideation or behaviour (some participants)	2 inpatients; 13 discharged
Sun et al. (2008)	To explore suicidal ex‐patients perceptions of the home environment and the provision of care in the home.	Taiwan	Interviews	Grounded theory approach	15	21–69	3 males; 12 females	Recent suicidal ideation or behaviour (all participants)	Discharged
Sun et al. (2009)	To explore suicidal ex‐patients perceptions of care provided at home following hospital discharge.	Taiwan	Interviews	Grounded theory approach	15	21–69	3 males; 12 females	Recent suicidal ideation or behaviour (all participants)	Discharged
Sun et al. (2012)	To explore and examine the home care provided by families to relatives who had recently been discharged from a hospital after suicide attempts.	Taiwan	Interviews	Grounded theory approach	30	n/a	10 males; 20 females	Recent suicidal ideation or behaviour (all participants)	Discharged
Tyler et al. (2021)	To investigate perceptions of safety in mental health transitions (hospital to community).	United Kingdom	Open‐ended questionnaire	Thematic analysis	27	n/a	n/a	Indication of suicidality in results section	n/a
Vandewalle et al. (2021)	To understand how patients in suicidal crises perceive their engagement with nurses in psychiatric hospitals.	Belgium	Interviews	Grounded theory approach	11	n/a	5 males; 6 females	Experienced suicidal ideation/behaviour in past year (all participants)	Inpatients
Wright et al. (2016)	To explore the nature of service user involvement in the admission and discharge process into and out of acute inpatient mental health care.	United Kingdom	Focus group	Thematic analysis	n/a	n/a	n/a	Indication of suicidality in results section	Discharged

^a^ Data missing for two participants.

### 3.3 Quality Assessment

Results of the quality assessment (CASP 2018) are presented in Appendix B. Consistent strengths were observed in the clear statement of research aims (CASP Q1), methodological appropriateness (CASP Q2) and alignment of research design with aims (CASP Q3), each achieved by 27/27 studies. However, notable weaknesses were identified in several areas. The researcher‐participant relationship (CASP Q6) was adequately addressed in only 2/27 studies, with many studies describing no relevant considerations at all or only brief and generic reflexivity statements. Similarly, ethical considerations (CASP Q7) were insufficiently detailed for 15/27 studies, often limited to mentions of ethical approval and superficial discussions of consent and confidentiality. Finally, 7/27 studies lacked sufficient rigour in data analysis (CASP Q8) or failed to provide adequate information to assess the analytic rigour.

### 3.4 Thematic Meta‐Synthesis

Overall, five analytic themes were developed, with each theme consisting of two descriptive themes (see Appendix C for a table of themes and additional example quotes to those below). The five analytic themes were as follows: (1) Feeling prepared for the transition home, (2) Returning from safety to everyday hardship, (3) The need for connection and understanding, (4) Feeling neglected by the system, (5) Taking the reins on recovery. Theme 1 focuses on the discharge process, often initiated by ward staff, and the difficult emotions that surround this. Theme 2 focuses more closely on the period prior to and immediately following discharge and describes the contrast in experience between the safety of the hospital ward and difficult lives in the community. Theme 3 highlights the importance of connection with others whilst on the ward and following discharge, and the resulting changes in how participants view themselves, and how others view them, as a result of their hospital stay. Theme 4 describes the types of support that participants feel they need following discharge, and how these needs are often not met. Finally, Theme 5 describes the need for participants to feel empowered to tackle their difficulties.

For each theme, first‐order accounts (i.e., participants' direct quotes) are presented in quotation marks and italics, whereas second‐order accounts (i.e., the study authors' interpretations) are presented in quotation marks but not italics.

### 3.5 Feeling Prepared for the Transition Home

#### 3.5.1 Discharge Planning

Participants described the importance of feeling psychologically, emotionally and practically prepared for being discharged from the hospital into the community. When discharge felt rushed and unpredictable, and when participants were not involved in the decision‐making process, this had a detrimental impact on their mental health. In most cases, discharge appeared undeniably rushed. One participant described how feeling unprepared triggered a suicide attempt ‘*To be notified about discharge on the same day is like hitting the pavement at 100km per hour. I was discharged without being prepared, and I became very confused and even more of a danger to myself. If I am not worthy enough of getting help from mental health care, then there is nothing more to do for me; my suicidal thoughts turn active, and I have tried committing suicide*’. (Berg et al. 2020). Others also described being given less than a days' notice, ‘*I was told “I want to send you home today”. Out of nowhere …*’ (Wright et al. 2016); ‘*The word discharge wasn't actually said until the end of the sentence on the last day’* (Cutcliffe et al. 2012b) and another described ‘*being pulled out of the blue* to be told *right you can go*’ (Coffey et al. 2019). In other cases, it was unclear how much notice of discharge participants had been given but it was clear that they felt uninvolved in the final decision, ‘One service user describes *not being ready to leave but not having a choice whether to be discharged or not*’ (Tyler et al. 2021). Involving service users in the discharge process and providing them with knowledge of the discharge process, including how to access support following discharge, helped them ‘feel safer’ (Tyler et al. 2021) and ‘in control’ (Redding et al. 2017). Some participants described periods of home leave prior to discharge as ‘useful’ (Owen‐Smith et al. 2014).

#### 3.5.2 Emotions and Coping Around Discharge

Discharge was often described as provoking a range of negative or difficult emotions, including anxiety, confusion, uncertainty, depression, hopelessness and shame. Importantly, patients' attitudes towards discharge appeared relevant, with ‘those who had not wanted to be discharged … reporting having experienced feelings of despair as soon as they arrived home’ (Owen‐Smith et al. 2014). Some participants left hospital feeling their suicidality had not been ‘*settled* or truly *worked out*’ leaving them with ‘a multitude of concerns and (unanswered) questions: Who (where) can I turn to? What am I going to do? Where do I go for help?… What can I do to help me endure/survive/get through this?’ (Cutcliffe et al. 2012b). Some described how suicide remained an option for them ‘on the very day that they were discharged’ (Cutcliffe et al. 2012b). Similarly, mental health issues were often ongoing following discharge, despite the inpatient stay, contrasting with some participants' expectations: ‘Following discharge, women tended to expect that recovery would be linear and progressive. However, depression frequently followed the acute episode, and milder periods of mood swings, anxiety, and low mood were also common. *I just thought once I was out of hospital, and the really delusional bit had gone, I thought, I'm out of the woods, you know … yeah it's all going to be fine, and then actually the depression afterwards, the deep, deep depression afterwards, was just such a blow, such a double whammy*.’ (Heron et al. 2012).

Conversely, those who had experienced reduced feelings of hopelessness were able to re‐frame their experiences and feel more optimistic about life post‐discharge ‘*after winter comes spring*. [I have] *more hope for improvement*’ (Bahlmann et al. 2022). In addition, reducing mental health symptoms helped protect some participants against suicidality. For example, in the context of delusional beliefs: ‘Some participants, whom on admission believed they were dying from somatic collapse or about to be killed by persecutors, described suicide as a way to escape the pain and pre‐empt certain death. But at discharge, this belief had dissipated.’ (Fredriksen et al. 2020).

### 3.6 Returning From Safety to Everyday Hardship

#### 3.6.1 Hospital as a Bubble

Participants often experienced hospital as a protective ‘bubble’ (Cutcliffe et al. 2012a; Owen‐Smith et al. 2014) or ‘safe haven … where they were free from imminent threat of suicide’ (Cutcliffe et al. 2012a; Fu et al. 2024), a place of comfort where their basic needs were being met. One participant said *‘It was nice, cause you're stuck in a room, and you have a nice air‐conditioned building, you have three meals a day* …’ and as a result of having ‘one's daily needs met … and … spared the vagaries of “the outside world”’ participants experienced ‘less suicidal ideation’ (Cutcliffe et al. 2012a). Having access to doctors and nurses and receiving 24‐h care contributed to feelings of safety and protection. Not all inpatient experiences were positive, one participant described ‘the experience of being hospitalised [as] a continuation of the trauma “*I thought I was this inanimate object that people needed to pathologise*”’ (though they later ‘came to view hospital as a sanctuary’, Jackson et al. 2020). Other participants found ‘closed doors and ward rules … negative if staff members were too rigid or just enforced them to demonstrate their power’ (Pelto‐Piri et al. 2019). Where hospital was viewed positively this contrasted with participants unsafe and unpredictable experiences in the community. Hospital admission helped reduce participants' levels of stress and despair by removing them from ‘intolerably stressful situations’ (Owen‐Smith et al. 2014). Being shielded from the outside world in this way meant that ‘some described death as no longer appearing to be their only alternative’ (Fredriksen et al. 2020). Understandably, however, participants worried that ‘*when re‐entering society, I may experience the same feelings and state I had before being hospitalized. I am concerned that I may still have thoughts of ending my life*’ (Fu et al. 2024).

#### 3.6.2 Difficult Lives in the Community

Following discharge, participants often returned to the same difficult lives that had contributed to them feeling suicidal on admission ‘*I had my old feeling, which was anxious, worrying again about what am I going to do?. .. just having to live again, you know get back on top of my bills, find a home, just face the real world again I think is what I was afraid of*’ (Cutcliffe et al. 2012b). Some participants described returning to lives that were worse than prior to their admission, such as ‘romantic relationships … damaged during the recent depressive episode’ (Fredriksen et al. 2020). The contrast between the support available in hospital and the lack of support in the community felt stark for some participants and the adjustment was difficult. Participants spoke of returning to lives that involved difficulties with finance, housing, and employment. Some spoke of how being discharged into a ‘*bad area*’ and around ‘*bad people*’ had a negative impact on their mental state (Brenisin et al. 2023). Drugs and alcohol were also more readily available in the community. Despite the difficulties that awaited many participants following discharge, some were happy to be out of hospital and to be ‘home sweet home’ (Redding et al. 2017).

### 3.7 The Need for Connection and Understanding

#### 3.7.1 Relationships With Others

Feeling isolated and misunderstood were common experiences for participants following discharge. Indeed, feeling ‘alone and unable to cope’ resulted in some participants abusing alcohol or ‘attempting suicide’ (Redding et al. 2017). Participants appeared stuck between desiring relationships and connection on the one hand, but ‘wishing to not have to face the world’ (Cutcliffe et al. 2012b) and finding interacting with others ‘*difficult*’ (Jackson et al. 2020) on the other hand. Even participants living with family reported feeling alone and suicidal. ‘*It's 4 o'clock in the morning and all your family's asleep, you're on your own, you're depressed, you may be suicidal, my first thought is I could do something now and no one could stop me*’ (Redding et al. 2017). In addition, participants were sometimes reluctant to speak to family about suicidal thoughts or distressing experiences due to feeling guilty for what they have ‘*already put them through*’ (Brenisin et al. 2023). Trust was key in allowing participants to discuss their suicide‐related difficulties with others.

The isolation experienced following discharge often contrasted with the connection that participants felt in hospital, and some described ‘missing the interaction with people within the hospital, the clients, and clinicians. Even in such somewhat artificial circumstances, the participants reported having some sense of comfort and therapeutic value in these interpersonal encounters and connections.’ (Cutcliffe et al. 2012b). Good relationships with staff often led to increased understanding of clients and their difficulties, which in some cases stopped the participants ‘from making a suicide attempt’ (Berg et al. 2020). Some participants preferred speaking to others with ‘lived experience’ (Hancock et al. 2022), rather than doctors and nurses, and felt ‘comradery because some of their suicidal thoughts were similar to mine’ (O'Connor et al. 2023). Some of these relationships with others with lived experience were maintained in the community and in some cases even appeared protective from suicide: ‘*If [fellow service user] hadn't of [sic] turned up there at the grave and took those tablets off me, I would have done it [attempted suicide] again*’ (Owen‐Smith et al. 2014).

#### 3.7.2 Identity and Stigma

Feelings of isolation were often compounded by experiences of stigmatisation due to being in hospital and a sense that other people did not understand them. Participants reported that ‘their experience of attempting suicide was not private, at least amongst family and friends… They experienced shame, embarrassment and increased social isolation’ (Jackson et al. 2020). Participants described loved ones feeling fear related to the suicide attempt and hospital admission: ‘*Fear of relapse and fear of me not sleeping, or having another dip … the ups and downs were just hideous for him … And also … because I did have two suicide attempts, and you know the fear for him of, “what is she going to do next”*’ (Heron et al. 2012). Some noticed shifts in their ‘personal identity’ due to ‘a change in their health status following their stay in hospital’ (Owen‐Smith et al. 2014). Relatedly, participants described the stress associated with the ongoing threat of feeling suicidal again and needing to return to hospital, like a ‘proverbial Sword of Damocles’ (Cutcliffe et al. 2012b), preventing them from rebuilding their lives and identity. Repeated admissions were potentially ‘detrimental’ to participants' mental state, leaving them feeling ‘like a failure’ (Brenisin et al. 2023), ‘which avert[ed] help‐seeking behaviour’ (Tyler et al. 2021).

### 3.8 Feeling Neglected by the System

#### 3.8.1 The Need for Accessible, Continuous and Timely Support After Discharge

The time period directly after discharge was described as extremely difficult by many participants, and many described inadequate and inaccessible support during this period. The ‘sudden termination of support’ (Owen‐Smith et al. 2014) that many participants experienced following discharge meant some participants felt ‘disenfranchised’ (Owen‐Smith et al. 2014), ‘vulnerab[le]’ (Owen‐Smith et al. 2014) and ‘unsupported’ (Cutcliffe et al. 2012b). Timing and duration of support offered appeared particularly important to participants, with very brief contact with professionals of ‘*5, 10 min*’ seeming ‘*a bit pointless*’ (referring to a post‐discharge home visit; Owen‐Smith et al. 2014). Broken promises made in care plans frustrated participants and contributed to them feeling ‘*really depressed again*’ (Owen‐Smith et al. 2014). Some participants had recognised that support was only offered on those occasions when they had made a suicide attempt, rather than on each occasion when they needed it, creating some confusion and suggesting a lack of preventative care being offered.

In contrast, when participants received support that was appropriate for their needs and a continuation of the care received on the wards, this was beneficial for their mental health and suicide risk. One way this was achieved was ‘through follow‐up plans or assigned/named members of staff’ (Tyler et al. 2021). Being able to contact someone 24‐h a day ‘was generally felt to be reassuring’ (Owen‐Smith et al. 2014) and ‘increased their feeling of security’ (Samuelsson et al. 2000). Similarly, participants who received a group suicide‐prevention intervention directly following discharge indicated ‘that the timing of the group was ideally situated to support the recovery process following a suicidal crisis’ (O'Connor et al. 2023). Importantly, participants felt a comprehensive care package should be developed and offered on the ward and then continued into the community ‘(e.g., psychotherapy, medications, rest, isolation, having a strict daily structure, group therapy and activities) … When these issues were not addressed, the participants experienced being a great risk to themselves after discharge’ (Berg et al. 2020). Some participants appeared to benefit from continuing contact with particular members of staff across the pre‐ to post‐discharge year, such as a peer worker, and found it unhelpful to ‘*see a whole load of different practitioners who invariably haven't read your notes and don't know your case history*’ (Tyler et al. 2021). Participants also benefited from help with community engagement following discharge, including help identifying ‘appropriate support services available for them in the community’ (Brenisin et al. 2023), and ‘support to improve social confidence’ (Awenat et al. 2018), as ‘returning to normal living with something that needed to be handled delicately’ (Redding et al. 2017).

#### 3.8.2 Desire for Human Care

Participants also described the negative impact of a lack of genuine, human, personalised care for their mental health. When participants had developed relationships with staff and felt safe, cared for, and understood, this helped reduce suicide risk. Participants reported feeling their experiences were dismissed by staff. One participant having a ‘*very bad night*’ felt invalidated after she was told by a nurse that ‘*it goes up and down for all of us in life, you know*’ (Hagen et al. 2018). Some participants received letters as part of a discharge package but ‘*the letters were impersonal, like round robins. To me, they suggested that you'd written a draft, it was printed off on the computer, and sent to everyone. … If you're looking for a way of reducing self‐harm or suicide after a hospital admission you need to have a sense of love. Don't you agree? You need to have this sense of actual human compassion instead of this computerized letter and a round robin of telephone numbers*’ (Bennewith et al. 2014). This desire to feel loved, to matter, or for someone to show they ‘*actually* [do] *care*’ (Bennewith et al. 2014) was common amongst participants. Again, feelings of unworthiness and burdensomeness were described by participants who ‘felt as though they were a drain on services’ (Redding et al. 2017) and chose not to discuss their experiences with family as they felt their ‘problems would be a burden to them if I talked to them’ (Cutcliffe et al. 2012a). Participants were more likely to accept support if this was genuinely emphasised by staff or if staff initiated the support, rather than participants needing to initiate the contact. Some described how engaging in purposeful and rewarding activities such as swimming or dancing in the community reduced their suicide risk. These activities sometimes provided the ‘love and care’ (Fu et al. 2024) from others that they desired.

### 3.9 Taking the Reins on Recovery

#### 3.9.1 Autonomy and Control

Being admitted to a mental health ward was understandably experienced by some as a loss of independence. Some felt the loss of independence was ‘*needed … at that time*’ (Cutcliffe et al. 2012a) and they recognised that they were ‘a danger to [them]selves’ (Wright et al. 2016). Some participants described discharge ‘as something that was done to them’ (Redding et al. 2017), rather than being a collaborative process, and this left them feeling powerless. Similarly, some felt powerless in relation to other members of staff, such as doctors. They felt their views around discharge were not considered but they questioned ‘*who was I to argue with the doctor you know?*’ (Redding et al. 2017). Participants expressed a desire to recognise when they were feeling suicidal and ‘perhaps be able to do something about it’ (Awenat et al. 2018), but when participants felt powerless, they lacked the confidence needed to respond to suicidal thoughts. Some participants appeared to describe a negative cycle of suicide attempts and increased dependence on others: ‘*My husband takes care of me so much. I was admitted to hospitals 22 times because of my depression and suicidal problems. He never leaves me. He stays with me all the time. I know that he's always observing me. Watching everything I do*.’ (Sun et al. 2009). Overall, feeling empowered to make change and regaining independence appeared important for reducing mental health difficulties and associated suicidality.

#### 3.9.2 Responsibility and Self‐Development

Once participants felt empowered to make changes it allowed them to take control over their own recovery, with appropriate support from services and family members. This initially involved participants being ‘honest with themselves’ (Brenisin et al. 2023)—accepting and understanding their difficulties and taking action needed to reduce suicidality. Participants recognised ‘that there was no magic wand and at some point they realised that they had to take responsibility for their recovery*: you've got to do a lot yourself erm, it's got to come from you really. You've got to want to get better, it's got to come from you*.’ (Redding et al. 2017). Taking control over recovery manifested in different ways, some participants read relevant books (e.g., about ‘*emotion self‐help*’; Fu et al. 2024), others practised self‐soothing (Cutcliffe et al. 2012b), ‘stay[ed] busy’ (Cutcliffe et al. 2012b), practised gratitude (Hagen et al. 2018), developed the ability to talk openly about personal issues (Hagen et al. 2018), or adhered to prescribed medication (e.g., Redding et al. 2017). Engaging with the local community and developing a ‘proper [support] network’ (Hagen et al. 2018) appeared helpful in reducing participants suicide risk. Importantly, participants found these social networks most helpful when they had a strong intention to change, rather than ‘expecting other people to do it for’ them (Redding et al. 2017). Sometimes taking control over recovery manifested as a battle where mental health difficulties and suicidality were viewed as something participants needed to ‘*take … on*’ (Redding et al. 2017). Whilst some participants found family support invaluable for recovery, the quality of the family relationship was important, as some participants felt family members were there to ‘*control* them rather than *care* for them’ (Sun et al. 2009).

## 4 Discussion

This study was the first to systematically review and meta‐synthesise qualitative research on people's experiences of suicidality prior to, and following, discharge from a mental health inpatient unit. Five analytic themes were identified from 29 studies that explored the experiences of current or previous inpatients. Themes suggested one possible reason for an increase in suicide risk during this period could be due to people being discharged home into difficult circumstances (e.g., relating to finances or housing) with little prior warning or support to prepare from the hospital staff. These difficult life circumstances may have contributed to suicidal thoughts or behaviours prior to being admitted to hospital, so it is unsurprising that a return to such circumstances could increase suicide risk. The themes also identified that support offered to people in this post‐discharge period was perceived, by some, as inadequate and that support appears protective against suicidality when person‐centred and extended into the community at a frequency and intensity tailored to the needs of the service user. This meta‐synthesis also identified that some participants found secure and sustained relationships protected them from suicide, sometimes directly (e.g., by removing the means to die by suicide) and other times indirectly (e.g., by helping them to feel more comfortable and less lonely). Participants who lacked connection with other people described feeling low and suicidal. Another theme reflected the mental health benefits of developing personal agency and control over recovery. Together, these findings demonstrate the cumulative impact of various factors, spanning from inpatient admission to community discharge, on participants' risk of suicide.

When considered alongside research into suicide risk factors, these findings highlight how the unique experiences of being discharged from a mental health inpatient unit could contribute to an increase in suicide risk. For example, experiences of burdensomeness, lack of social support, hopelessness, and depression were described by participants in the included studies, and all have been proposed as relevant suicide mechanisms (O'Connor and Nock 2014). Feelings of burdensomeness were provoked when services were not responsive to participants' post‐discharge support needs, or towards family members who participants felt had already suffered too much when they attempted to end their lives. Participants also described feelings of isolation and lacking social support being compounded by the hospital admission, which damaged relationships and increased perceived stigma towards them. Hopelessness and depression increased in some participants who, contrary to their expectations, found that their mental health problems (including suicidality) were not resolved whilst in hospital. Being discharged back into the same lives that had contributed to their suicidality also increased this sense of hopelessness.

A surprising finding was the positive way many participants described their time in hospital, as reflected in the second analytic theme, ‘Returning from safety to everyday hardship’. Hospital was described as a place of safety and protection. Participants also found the hospital provided opportunities to connect with others and ensured basic needs, such as food and warmth, were met. This contrasts with other research that describes more negative experiences of inpatient life. For example, a review of 72 studies investigating experiences of inpatient mental health services found that participants often described feeling unsafe, as they feared being harmed whilst being restrained by staff or by other patients (Staniszewska et al. 2019). Due to this fear of others, participants often spent time in their rooms or absconded from the ward, meaning they did not develop the kind of helpful relationships discussed in the current review (Staniszewska et al. 2019). Participants also described feeling bored on inpatient wards due to a lack of ward activities and felt the wards were overcrowded (Staniszewska et al. 2019). One possible explanation for these differences in findings is that the studies in the current review involved participants discussing hospital in contrast to their lives in the community. The positive aspects of the ward may have therefore been enhanced, and the negative aspects reduced, when discussed in the context of their difficult lives outside of hospital.

### 4.1 Clinical Implications and Future Research

The findings of this review have several implications for clinical practice and further work. As a broad recommendation, interventions that target the reduction of hopelessness and increase service‐users' participation in aftercare are likely to reduce suicide risk, as these mechanisms were identified in this qualitative review, as well as existing quantitative reviews (Large et al. 2011; O'Connell et al. 2021; Tai et al. 2025). Other recommendations are more tentative as they are based mostly on the findings of this review. These recommendations should therefore be evaluated, where possible, such as within a clinical trial. These recommendations include service‐users being provided advanced warning of discharge and their genuine involvement in the discharge process from as early as possible. Where possible, a plan should be made to tackle external stressors (e.g., financial) whilst service‐users are still on wards to help ease the transition from hospital back to the community. Similarly, support should be readily available and particularly intensive in the first few days and weeks following discharge when participants may be struggling to adjust to life outside of hospital. Efforts should be placed on establishing trusting, meaningful relationships between service‐users and staff, and service‐users and other people within their community, to ensure participants feel a sense of connection and belonging, as this may protect them from suicide. Relationships with staff, where possible, should be continued into the community to ensure that participants feel well supported by somebody they already have an established relationship with. This support may be in the form of a specialised team who work both on the wards and in the community and who focus on supporting service‐users in their transition home. Following discharge, staff should gradually work with service‐users to develop independence. This work could be multi‐faceted and could include helping participants develop hobbies or interests in the community, encouraging the consumption of relevant psychoeducation, attending therapy to develop self‐awareness and knowledge of mental health problems, and positive encouragement when participants demonstrate such independence. Importantly, independence in this context does *not* mean that service users should be expected to live and cope alone without the support of others. Rather, service users should be supported to develop an increased self‐confidence in their ability to manage their suicidality should it arise again, which could include seeking help from friends, family, or services, where necessary. Psychological therapy that begins in the inpatient setting and continues into the community could help service‐users develop their independence and self‐awareness, and such interventions appear both desirable and acceptable to service‐users (e.g., Awenat et al. 2018; Haddock et al. 2019).

Interestingly, many of these recommendations overlap with pre‐existing guidelines on how to support service‐users in the transition from inpatient wards to the community. For example, guidance produced by the National Institute for Health and Care Excellence in the United Kingdom highlights the importance of person‐centred, continuous care that puts the service user at the centre of decision making, including around discharge (NICE 2016). The extent to which this specific advice is implemented is unclear; however, as similar guidelines do not appear to be well adhered to (e.g., guidelines in the United Kingdom for schizophrenia, Berry and Haddock 2008; guidelines in Germany for depression/suicide, Teismann et al. 2022). The reasons such guidelines are not adhered to is likely due to a combination of staff, service‐user, and organisational factors (Berry and Haddock 2008). Staff factors may include lack of awareness of the guidance (perhaps due to poor dissemination), or they may not feel confident or competent in implementing it. Service user factors include a lack of engagement in the discharge process or in decision‐making, even if the opportunity is available to them (e.g., due to feeling disempowered or hopeless or the opposite, feeling the crisis has passed and they no longer need to engage in the discharge process). However, the findings from this review suggest that is not the case for at least some service users. Organisational factors include the lack of training and resources available for staff to implement the guidelines as intended. Future studies could explore these ideas further by investigating whether mental health hospital discharge guidelines are being followed in a variety of countries, including the barriers to implementation. Interventions could be designed and evaluated that address the staff, service user and organisational factors described above, and any other barriers to implementation that are identified. For example, does offering staff further training on the guidelines improve how well they are implemented?

### 4.2 Limitations

There were several strengths to this study which was the first meta‐synthesis on the topic and summarised the experiences of over 400 participants across 11 countries. The results have highlighted several areas where mental health services, and future research studies, can focus to reduce suicide amongst inpatients and those recently discharged from hospital. However, there were also several limitations to the study. First, studies varied in how focussed they were on the experiences of suicidality, and it was not always clear from study reports if themes or quotes were referring specifically to the risk of suicide or to broader issues that may be indirectly related to suicide, such as poor mental health, hopelessness or poor quality of life. However, this is reflective of the literature included in the systematic review, where the link between specific experiences and suicide was not always clear (even when this was a clearly stated aim of the study), suggesting this may be a broader issue with qualitative research in this area. Second, some of the studies focussed on the discharge period in current inpatients who may not have experienced discharge before. Whilst the views of such participants are valuable in answering the research questions, they provide a different kind of perspective to participants who have already been discharged. Third, the results of this study are inevitably biased in that participants who experienced the greatest risk of suicide—that is, those who died by suicide—could not be interviewed about their experiences. Similarly, included participants may have biased towards having more positive experiences than the average inpatient (e.g., because they were happy to collaborate with researchers linked to the ward, discussing positive experiences is likely less distressing than sharing negative experiences) or towards those with more negative experiences than average (i.e., because they wanted their concerns to be heard, or were hoping to make positive changes to the inpatient setting). As a result, the full breadth of experiences may not be captured by the review.

### 4.3 Conclusions

The findings of this review highlighted the period of discharge from a mental health hospital as a frightening, confusing and disempowering experience for many participants. Whilst experiences varied, participants from around the world perceived support as inadequate in the post‐discharge period. Post‐discharge suicides may be prevented through involving service‐users in decision making around their post‐discharge support, supporting them in developing meaningful connections with others, and encouraging them to develop independence around their recovery, and research should be conducted to assess these suggestions.

## Author Contributions

Dr. Connor Heapy designed the study, conducted the literature searches, analysed the data and wrote the first draft of the manuscript. Professors Daniel Pratt and Gillian Haddock were supervisors on the project and contributed to study design, providing feedback on several drafts of the manuscript, and have approved the final manuscript. Miss Jordan Parkinson served as a second rater for title/abstract screening, full‐text screening and quality assessment and provided comments on a final draft of the paper.

## Funding

The authors have nothing to report.

## Conflicts of Interest

The authors declare no conflicts of interest.

## Supporting information


**Data S1:** Appendix A: Search terms.

**Data S2:** Appendix B: CASP quality appraisal ratings.

**Data S3:** Appendix C: Table of analytic and descriptive themes with additional quotes.

## Data Availability

Data sharing not applicable to this article as no datasets were generated or analysed during the current study.
