# Supplementary material for: Experiences of Suicidality Following Discharge From a Mental Health Inpatient Unit: A Systematic Review and Meta‐Synthesis
Source: Clin Psychol Psychother. 2026 Feb 11;33(1):e70234. doi: 10.1002/cpp.70234 (PMC12892015; doi:10.1002/cpp.70234)
Supplement: Supplementary file 1 — Data S1: Appendix A: Search terms. [file CPP-33-e70234-s001.docx]

**Appendix A: Database search terms**

**PsycINFO search terms**

1 adults.mp. [mp=title, abstract, heading word, table of contents, key concepts, original title, tests & measures, mesh word]

2 service-users.mp. [mp=title, abstract, heading word, table of contents, key concepts, original title, tests & measures, mesh word]

3 "service users".mp. [mp=title, abstract, heading word, table of contents, key concepts, original title, tests & measures, mesh word]

4 patients.mp. [mp=title, abstract, heading word, table of contents, key concepts, original title, tests & measures, mesh word]

5 inpatient*.mp. [mp=title, abstract, heading word, table of contents, key concepts, original title, tests & measures, mesh word]

6 in-patient*.mp. [mp=title, abstract, heading word, table of contents, key concepts, original title, tests & measures, mesh word]

7 suicid*.mp. [mp=title, abstract, heading word, table of contents, key concepts, original title, tests & measures, mesh word]

8 "self harm*".mp. [mp=title, abstract, heading word, table of contents, key concepts, original title, tests & measures, mesh word]

9 self-harm*.mp. [mp=title, abstract, heading word, table of contents, key concepts, original title, tests & measures, mesh word]

10 self-injur*.mp. [mp=title, abstract, heading word, table of contents, key concepts, original title, tests & measures, mesh word]

11 "self injur*".mp. [mp=title, abstract, heading word, table of contents, key concepts, original title, tests & measures, mesh word]

12 self-poison*.mp. [mp=title, abstract, heading word, table of contents, key concepts, original title, tests & measures, mesh word]

13 "self poison*".mp. [mp=title, abstract, heading word, table of contents, key concepts, original title, tests & measures, mesh word]

14 overdos*.mp. [mp=title, abstract, heading word, table of contents, key concepts, original title, tests & measures, mesh word]

15 "feeling unsafe".mp. [mp=title, abstract, heading word, table of contents, key concepts, original title, tests & measures, mesh word]

16 "feeling safe".mp. [mp=title, abstract, heading word, table of contents, key concepts, original title, tests & measures, mesh word]

17 discharge.mp. [mp=title, abstract, heading word, table of contents, key concepts, original title, tests & measures, mesh word]

18 postdischarge.mp. [mp=title, abstract, heading word, table of contents, key concepts, original title, tests & measures, mesh word]

19 post-discharge.mp. [mp=title, abstract, heading word, table of contents, key concepts, original title, tests & measures, mesh word]

20 "psychiatric ward".mp. [mp=title, abstract, heading word, table of contents, key concepts, original title, tests & measures, mesh word]

21 "psychiatric unit".mp. [mp=title, abstract, heading word, table of contents, key concepts, original title, tests & measures, mesh word]

22 "psychiatric hospital*".mp. [mp=title, abstract, heading word, table of contents, key concepts, original title, tests & measures, mesh word]

23 "mental health ward".mp. [mp=title, abstract, heading word, table of contents, key concepts, original title, tests & measures, mesh word]

24 "mental health hospital*".mp. [mp=title, abstract, heading word, table of contents, key concepts, original title, tests & measures, mesh word]

25 "psychiatric inpatient*".mp. [mp=title, abstract, heading word, table of contents, key concepts, original title, tests & measures, mesh word]

26 "mental health unit".mp. [mp=title, abstract, heading word, table of contents, key concepts, original title, tests & measures, mesh word]

27 qualitative.mp. [mp=title, abstract, heading word, table of contents, key concepts, original title, tests & measures, mesh word]

28 experience*.mp. [mp=title, abstract, heading word, table of contents, key concepts, original title, tests & measures, mesh word]

29 perspect*.mp. [mp=title, abstract, heading word, table of contents, key concepts, original title, tests & measures, mesh word]

30 interview.mp. [mp=title, abstract, heading word, table of contents, key concepts, original title, tests & measures, mesh word]

31 "focus group".mp. [mp=title, abstract, heading word, table of contents, key concepts, original title, tests & measures, mesh word]

32 user-views.mp. [mp=title, abstract, heading word, table of contents, key concepts, original title, tests & measures, mesh word]

33 "thematic analysis".mp. [mp=title, abstract, heading word, table of contents, key concepts, original title, tests & measures, mesh word]

34 "interpretative phenomenological analysis".mp. [mp=title, abstract, heading word, table of contents, key concepts, original title, tests & measures, mesh word]

35 "content analysis".mp. [mp=title, abstract, heading word, table of contents, key concepts, original title, tests & measures, mesh word]

36 1 or 2 or 3 or 4 or 5 or 6

37 7 or 8 or 9 or 10 or 11 or 12 or 13 or 14 or 15 or 16

38 17 or 18 or 19 or 20 or 21 or 22 or 23 or 24 or 25 or 26

39 27 or 28 or 29 or 30 or 31 or 32 or 33 or 34 or 35

40 36 and 37 and 38 and 39

**MEDLINE search terms**

adults.mp. [mp=title, book title, abstract, original title, name of substance word, subject heading word, floating sub-heading word, keyword heading word, organism supplementary concept word, protocol supplementary concept word, rare disease supplementary concept word, unique identifier, synonyms, population supplementary concept word, anatomy supplementary concept word]

2 service-users.mp. [mp=title, book title, abstract, original title, name of substance word, subject heading word, floating sub-heading word, keyword heading word, organism supplementary concept word, protocol supplementary concept word, rare disease supplementary concept word, unique identifier, synonyms, population supplementary concept word, anatomy supplementary concept word]

3 "service users".mp. [mp=title, book title, abstract, original title, name of substance word, subject heading word, floating sub-heading word, keyword heading word, organism supplementary concept word, protocol supplementary concept word, rare disease supplementary concept word, unique identifier, synonyms, population supplementary concept word, anatomy supplementary concept word]

4 patients.mp. [mp=title, book title, abstract, original title, name of substance word, subject heading word, floating sub-heading word, keyword heading word, organism supplementary concept word, protocol supplementary concept word, rare disease supplementary concept word, unique identifier, synonyms, population supplementary concept word, anatomy supplementary concept word]

5 inpatient*.mp. [mp=title, book title, abstract, original title, name of substance word, subject heading word, floating sub-heading word, keyword heading word, organism supplementary concept word, protocol supplementary concept word, rare disease supplementary concept word, unique identifier, synonyms, population supplementary concept word, anatomy supplementary concept word]

6 in-patient*.mp. [mp=title, book title, abstract, original title, name of substance word, subject heading word, floating sub-heading word, keyword heading word, organism supplementary concept word, protocol supplementary concept word, rare disease supplementary concept word, unique identifier, synonyms, population supplementary concept word, anatomy supplementary concept word]

7 suicid*.mp. [mp=title, book title, abstract, original title, name of substance word, subject heading word, floating sub-heading word, keyword heading word, organism supplementary concept word, protocol supplementary concept word, rare disease supplementary concept word, unique identifier, synonyms, population supplementary concept word, anatomy supplementary concept word]

8 "self harm*".mp. [mp=title, book title, abstract, original title, name of substance word, subject heading word, floating sub-heading word, keyword heading word, organism supplementary concept word, protocol supplementary concept word, rare disease supplementary concept word, unique identifier, synonyms, population supplementary concept word, anatomy supplementary concept word]

9 self-harm*.mp. [mp=title, book title, abstract, original title, name of substance word, subject heading word, floating sub-heading word, keyword heading word, organism supplementary concept word, protocol supplementary concept word, rare disease supplementary concept word, unique identifier, synonyms, population supplementary concept word, anatomy supplementary concept word]

10 self-injur*.mp. [mp=title, book title, abstract, original title, name of substance word, subject heading word, floating sub-heading word, keyword heading word, organism supplementary concept word, protocol supplementary concept word, rare disease supplementary concept word, unique identifier, synonyms, population supplementary concept word, anatomy supplementary concept word]

11 "self injur*".mp. [mp=title, book title, abstract, original title, name of substance word, subject heading word, floating sub-heading word, keyword heading word, organism supplementary concept word, protocol supplementary concept word, rare disease supplementary concept word, unique identifier, synonyms, population supplementary concept word, anatomy supplementary concept word]

12 self-poison*.mp. [mp=title, book title, abstract, original title, name of substance word, subject heading word, floating sub-heading word, keyword heading word, organism supplementary concept word, protocol supplementary concept word, rare disease supplementary concept word, unique identifier, synonyms, population supplementary concept word, anatomy supplementary concept word]

13 "self poison*".mp. [mp=title, book title, abstract, original title, name of substance word, subject heading word, floating sub-heading word, keyword heading word, organism supplementary concept word, protocol supplementary concept word, rare disease supplementary concept word, unique identifier, synonyms, population supplementary concept word, anatomy supplementary concept word]

14 overdos*.mp. [mp=title, book title, abstract, original title, name of substance word, subject heading word, floating sub-heading word, keyword heading word, organism supplementary concept word, protocol supplementary concept word, rare disease supplementary concept word, unique identifier, synonyms, population supplementary concept word, anatomy supplementary concept word]

15 "feeling unsafe".mp. [mp=title, book title, abstract, original title, name of substance word, subject heading word, floating sub-heading word, keyword heading word, organism supplementary concept word, protocol supplementary concept word, rare disease supplementary concept word, unique identifier, synonyms, population supplementary concept word, anatomy supplementary concept word]

16 "feeling safe".mp. [mp=title, book title, abstract, original title, name of substance word, subject heading word, floating sub-heading word, keyword heading word, organism supplementary concept word, protocol supplementary concept word, rare disease supplementary concept word, unique identifier, synonyms, population supplementary concept word, anatomy supplementary concept word]

17 discharge.mp. [mp=title, book title, abstract, original title, name of substance word, subject heading word, floating sub-heading word, keyword heading word, organism supplementary concept word, protocol supplementary concept word, rare disease supplementary concept word, unique identifier, synonyms, population supplementary concept word, anatomy supplementary concept word]

18 postdischarge.mp. [mp=title, book title, abstract, original title, name of substance word, subject heading word, floating sub-heading word, keyword heading word, organism supplementary concept word, protocol supplementary concept word, rare disease supplementary concept word, unique identifier, synonyms, population supplementary concept word, anatomy supplementary concept word]

19 post-discharge.mp. [mp=title, book title, abstract, original title, name of substance word, subject heading word, floating sub-heading word, keyword heading word, organism supplementary concept word, protocol supplementary concept word, rare disease supplementary concept word, unique identifier, synonyms, population supplementary concept word, anatomy supplementary concept word]

20 "psychiatric ward".mp. [mp=title, book title, abstract, original title, name of substance word, subject heading word, floating sub-heading word, keyword heading word, organism supplementary concept word, protocol supplementary concept word, rare disease supplementary concept word, unique identifier, synonyms, population supplementary concept word, anatomy supplementary concept word]

21 "psychiatric unit".mp. [mp=title, book title, abstract, original title, name of substance word, subject heading word, floating sub-heading word, keyword heading word, organism supplementary concept word, protocol supplementary concept word, rare disease supplementary concept word, unique identifier, synonyms, population supplementary concept word, anatomy supplementary concept word]

22 "psychiatric hospital*".mp. [mp=title, book title, abstract, original title, name of substance word, subject heading word, floating sub-heading word, keyword heading word, organism supplementary concept word, protocol supplementary concept word, rare disease supplementary concept word, unique identifier, synonyms, population supplementary concept word, anatomy supplementary concept word]

23 "mental health ward".mp. [mp=title, book title, abstract, original title, name of substance word, subject heading word, floating sub-heading word, keyword heading word, organism supplementary concept word, protocol supplementary concept word, rare disease supplementary concept word, unique identifier, synonyms, population supplementary concept word, anatomy supplementary concept word]

24 "mental health hospital*".mp. [mp=title, book title, abstract, original title, name of substance word, subject heading word, floating sub-heading word, keyword heading word, organism supplementary concept word, protocol supplementary concept word, rare disease supplementary concept word, unique identifier, synonyms, population supplementary concept word, anatomy supplementary concept word]

25 "psychiatric inpatient*".mp. [mp=title, book title, abstract, original title, name of substance word, subject heading word, floating sub-heading word, keyword heading word, organism supplementary concept word, protocol supplementary concept word, rare disease supplementary concept word, unique identifier, synonyms, population supplementary concept word, anatomy supplementary concept word]

26 "mental health unit".mp. [mp=title, book title, abstract, original title, name of substance word, subject heading word, floating sub-heading word, keyword heading word, organism supplementary concept word, protocol supplementary concept word, rare disease supplementary concept word, unique identifier, synonyms, population supplementary concept word, anatomy supplementary concept word]

27 qualitative.mp. [mp=title, book title, abstract, original title, name of substance word, subject heading word, floating sub-heading word, keyword heading word, organism supplementary concept word, protocol supplementary concept word, rare disease supplementary concept word, unique identifier, synonyms, population supplementary concept word, anatomy supplementary concept word]

28 experience*.mp. [mp=title, book title, abstract, original title, name of substance word, subject heading word, floating sub-heading word, keyword heading word, organism supplementary concept word, protocol supplementary concept word, rare disease supplementary concept word, unique identifier, synonyms, population supplementary concept word, anatomy supplementary concept word]

29 perspect*.mp. [mp=title, book title, abstract, original title, name of substance word, subject heading word, floating sub-heading word, keyword heading word, organism supplementary concept word, protocol supplementary concept word, rare disease supplementary concept word, unique identifier, synonyms, population supplementary concept word, anatomy supplementary concept word]

30 interview.mp. [mp=title, book title, abstract, original title, name of substance word, subject heading word, floating sub-heading word, keyword heading word, organism supplementary concept word, protocol supplementary concept word, rare disease supplementary concept word, unique identifier, synonyms, population supplementary concept word, anatomy supplementary concept word]

31 "focus group".mp. [mp=title, book title, abstract, original title, name of substance word, subject heading word, floating sub-heading word, keyword heading word, organism supplementary concept word, protocol supplementary concept word, rare disease supplementary concept word, unique identifier, synonyms, population supplementary concept word, anatomy supplementary concept word]

32 user-views.mp. [mp=title, book title, abstract, original title, name of substance word, subject heading word, floating sub-heading word, keyword heading word, organism supplementary concept word, protocol supplementary concept word, rare disease supplementary concept word, unique identifier, synonyms, population supplementary concept word, anatomy supplementary concept word]

33 "thematic analysis".mp. [mp=title, book title, abstract, original title, name of substance word, subject heading word, floating sub-heading word, keyword heading word, organism supplementary concept word, protocol supplementary concept word, rare disease supplementary concept word, unique identifier, synonyms, population supplementary concept word, anatomy supplementary concept word]

34 "interpretative phenomenological analysis".mp. [mp=title, book title, abstract, original title, name of substance word, subject heading word, floating sub-heading word, keyword heading word, organism supplementary concept word, protocol supplementary concept word, rare disease supplementary concept word, unique identifier, synonyms, population supplementary concept word, anatomy supplementary concept word]

35 "content analysis".mp. [mp=title, book title, abstract, original title, name of substance word, subject heading word, floating sub-heading word, keyword heading word, organism supplementary concept word, protocol supplementary concept word, rare disease supplementary concept word, unique identifier, synonyms, population supplementary concept word, anatomy supplementary concept word]

36 1 or 2 or 3 or 4 or 5 or 6

37 7 or 8 or 9 or 10 or 11 or 12 or 13 or 14 or 15 or 16

38 17 or 18 or 19 or 20 or 21 or 22 or 23 or 24 or 25 or 26

39 27 or 28 or 29 or 30 or 31 or 32 or 33 or 34 or 35

40 36 and 37 and 38 and 39

**Web of Science (including ProQuest) search terms**

1: TS=(adults OR service-users OR “service users” OR patients OR inpatient* OR in-patient*)

2: TS=(suicid* OR “self harm*” OR self-harm* OR self-injur* OR “self injur*” OR self-poison* OR “self poison*” OR overdos* OR “feeling unsafe” OR “feeling safe”)

3: TS=(discharge OR postdischarge OR post-discharge OR “psychiatric ward” OR “psychiatric unit” OR “psychiatric hospital*” OR “mental health ward” OR “mental health unit” OR “mental health hospital*” OR "psychiatric inpatient*")

4: TS=(qualitative OR experience* OR perspect* OR interview OR “focus group” OR user-views OR “thematic analysis” OR “interpretative phenomenological analysis” OR “content analysis”)

5: #1 AND #2 AND #3 AND #

**PubMed search terms**

1 adults[Text Word]

2 service-users[Text Word]

3 "service users"[Text Word]

4 patients[Text Word]

5 inpatient*[Text Word]

6 in-patient*[Text Word]

7 suicid*[Text Word]

8 "self harm*"[Text Word]

9 self-harm*[Text Word]

10 self-injur*[Text Word]

11 "self injur*"[Text Word]

12 self-poison*[Text Word]

13 "self poison*"[Text Word]

14 overdos*[Text Word]

15 "feeling unsafe"[Text Word]

16 "feeling safe"[Text Word]

17 discharge[Text Word]

18 postdischarge[Text Word]

19 post-discharge[Text Word]

20 "psychiatric ward"[Text Word]

21 "psychiatric unit"[Text Word]

22 "psychiatric hospital*"[Text Word]

23 "mental health ward"[Text Word]

24 "mental health unit"[Text Word]

25 "mental health hospital*"[Text Word]

26 "psychiatric inpatient*"[Text Word]

27 qualitative[Text Word]

28 experience*[Text Word]

29 perspect*[Text Word]

30 interview[Text Word]

31 "focus group"[Text Word]

32 user-views[Text Word]

33 "thematic analysis"[Text Word]

34 "interpretative phenomenological analysis"[Text Word]

35 "content analysis"[Text Word]

36 #1 OR #2 OR #3 OR #4 OR #5 OR #6

37 #7 OR #8 OR #9 OR #10 OR #11 OR #12 OR #13 OR #14 OR #15 OR #16

38 #17 OR #18 OR #19 OR #20 OR #21 OR #22 OR #23 OR #24 OR #25 OR #26

39 #27 OR #28 OR #29 OR #30 OR #31 OR #32 OR #33 OR #34 OR #35

40 #36 AND #37 AND #38 and #39
