# Supplementary material for: Experiences of Suicidality Following Discharge From a Mental Health Inpatient Unit: A Systematic Review and Meta‐Synthesis
Source: Clin Psychol Psychother. 2026 Feb 11;33(1):e70234. doi: 10.1002/cpp.70234 (PMC12892015; doi:10.1002/cpp.70234)
Supplement: Supplementary file 2 — Data S2: Appendix B: CASP quality appraisal ratings. [file CPP-33-e70234-s003.docx]

**Appendix B**

Table showing appraisal ratings for each study (using Critical Appraisal Skills Programme, CASP, Checklist, 2018)

| Study | Q1. Was there a clear statement of the aims of the research? | Q2. Is a qualitative methodology appropriate? | Q3. Was the research design appropriate to address the aims of the research? | Q4. Was the recruitment strategy appropriate to the aims of the research? | Q5. Was the data collected in a way that addressed the research issue? | Q6. Has the relationship between researcher and participants been adequately considered? | Q7. Have ethical issues been taken into consideration? | Q8. Was the data analysis sufficiently rigorous? | Q9. Is there a clear statement of findings? | Q10. Was the research valuable? |
| --- | --- | --- | --- | --- | --- | --- | --- | --- | --- | --- |
| Awenat et al. (2018) | Yes | Yes | Yes | Yes | Yes | Yes | Yes | Yes | Yes | Yes |
| Bahlmann et al. (2022) | Yes | Yes | Yes | Yes | No | No | No | No | Yes | Yes |
| Bennewith et al. (2014) | Yes | Yes | Yes | Yes | Yes | No | No | No | No | Yes |
| Berg et al. (2020) | Yes | Yes | Yes | Yes | Yes | No | Yes | Yes | Yes | Yes |
| Brenisin et al. (2023) | Yes | Yes | Yes | No | No | No | No | No | Yes | Yes |
| Chen et al. (2022) | Yes | Yes | Yes | Yes | Yes | No | No | Yes | Yes | Yes |
| Coffey et al. (2019) | Yes | Yes | Yes | Yes | Yes | No | No | Yes | Yes | Yes |
| Cutcliffe et al. (2012a) | Yes | Yes | Yes | Yes | Yes | No | No | Yes | Yes | Yes |
| Cutcliffe et al. (2012b) | Yes | Yes | Yes | Yes | Yes | No | No | Yes | Yes | Yes |
| Fredriksen et al. (2020) | Yes | Yes | Yes | Yes | Yes | Yes | No | Yes | Yes | Yes |
| Fu et al. (2024) | Yes | Yes | Yes | Yes | Yes | No | Yes | Yes | Yes | Yes |
| Ghio et al. (2011) | Yes | Yes | Yes | Yes | Yes | No | Yes | No | Yes | Yes |
| Hagen et al. (2018) | Yes | Yes | Yes | Yes | Yes | No | Yes | Yes | Yes | Yes |
| Hagen et al. (2020) | Yes | Yes | Yes | No | Yes | No | Yes | Yes | Yes | Yes |
| Hancock et al. (2022) | Yes | Yes | Yes | Yes | Yes | No | No | No | Yes | No |
| Heron et al. (2012) | Yes | Yes | Yes | Yes | Yes | No | No | Yes | Yes | Yes |
| Jackson et al. (2020) | Yes | Yes | Yes | No | Yes | No | Yes | Yes | Yes | Yes |
| O’Connor et al. (2021) | Yes | Yes | Yes | Yes | Can’t tell | No | No | No | Yes | Yes |
| Owen-smith et al. (2014)  Pelto-Piri et al. (2019) | Yes  Yes | Yes  Yes | Yes  Yes | Yes  Yes | Yes  Yes | No  No | No  No | Yes  Yes | Yes  Yes | Yes  Yes |
| Redding et al. (2017) | Yes | Yes | Yes | Yes | Can’t tell | Can’t tell | Can’t tell | Yes | Yes | Yes |
| Samuelsson et al. (2000) | Yes | Yes | Yes | Yes | Yes | No | Yes | Yes | Yes | Yes |
| Steinberg et al. (2024) | Yes | Yes | Yes | Yes | Yes | No | No | No | Yes | Yes |
| Sun et al. (2008) | Yes | Yes | Yes | Yes | Yes | No | Yes | Yes | Yes | Yes |
| Sun et al. (2009) | Yes | Yes | Yes | Yes | Yes | No | Yes | Yes | Yes | Yes |
| Sun et al. (2012) | Yes | Yes | Yes | Yes | Yes | No | Yes | Yes | Yes | Yes |
| Tyler et al. (2021) | Yes | Yes | Yes | Yes | Can’t tell | No | Yes | Yes | Yes | Yes |
| Vanderwall et al. (2021) | Yes | Yes | Yes | Yes | Yes | Can’t tell | Yes | Yes | Yes | Yes |
| Wright et al. (2015) | Yes | Yes | Yes | Can’t tell | Yes | No | Yes | Yes | Yes | Yes |
|  |  |  |  |  |  |  |  |  |  |  |

*Note.*

Yes = met criteria; Can’t tell = insufficient information to make a judgement; No = no evidence to suggest the criteria was met
