# Supplementary material for: Experiences of Suicidality Following Discharge From a Mental Health Inpatient Unit: A Systematic Review and Meta‐Synthesis
Source: Clin Psychol Psychother. 2026 Feb 11;33(1):e70234. doi: 10.1002/cpp.70234 (PMC12892015; doi:10.1002/cpp.70234)
Supplement: Supplementary file 3 — Data S3: Appendix C: Table of analytic and descriptive themes with additional quotes. [file CPP-33-e70234-s002.docx]

**Appendix C**

Table showing analytic and descriptive themes and example quotes

| Analytic theme | Descriptive theme | Example quotes |
| --- | --- | --- |
| 1. Feeling prepared for the transition home | - 1. Discharge planning | “*All four informants who had not wanted to be discharged said they had had urges to harm themselves since discharge (two had done so), and three reported experiencing suicidal feelings during this period.”* (Owen-Smith et al., 2014) |
|  |  | “*I know there are lots of times where I feel so overwhelmed from the discharge especially if I’m being discharged before I feel ready so it would be really helpful to have someone else there with me um while they’re going over the discharge planning and to leave with me um just so that there’s someone who is hearing it who can help me remember*.” (Cutcliffe et al., 2012b) |
|  | - 1. Emotions and coping around discharge | "*If one is relatively ungrown inside and still in a fragile mental state, any external change may stimulate a relapse of the disease*” (Fu et al., 2024) |
|  |  | “*I was uncomfortable after being discharged. My chest was tight, and I couldn’t breathe and wanted to die. One time, I tried to commit suicide by injecting air into a blood vessel with a syringe, but I didn’t know how to do it. I was sad, and the lifeline came to mind. I called them, and they helped me calm down and feel better*.” (Sun et al., 2012) |
| 1. Returning from safety to everyday hardship | - 1. Hospital as a bubble | “Several participants experienced it as helpful during admission to be physically distanced from stressors, demands and imagined persecutors from the outside world” (Fredriksen et al., 2020) |
|  |  | “Several people described the fear they had about leaving inpatient unit supports when discharged. SU13 said she felt: “*like I was a little bird . . . that’s been looked after and then they let it out of the cage . . . you’ve been nurtured in the hospital environment, then [they are] releasing you back into the wild*” (Hancock et al., 2022) |
|  | - 1. Difficult lives in the community | “*I was reasonably happy – well, very happy to be out of the hospital for the first 2 weeks, but … after being home for a couple of weeks now, it’s sunk in how much of a problem I have got ﬁnancially*” (Owen-Smith et al., 2014) |
|  |  | “*Just walking down the street is a challenge you know because you know, you tend to find, especially in the area that I live XX you tend to find a lot of people there are on the corners drinking, there’s a lot of people around that area that are either using or have been using psychiatric services. There’s a few erm, houses along the street I live that are dry house for alcoholics and drug addicts so the area is quite a um, colourful mix of people*.” (Redding et al., 2017) |
| 1. The need for connection and understanding | - 1. Relationships with others | “*I felt like I was going to miss a lot of the people, like I was going to miss you know the nurses and (nurse’s name) and you know Dr. (Dr’s name) and so forth because I felt like they were just like really nice warm people and I really enjoyed talking to them*.” (Cutcliffe et al., 2012a) |
|  |  | “*…a sense of disconnection between me and them, the family has never understood m*e” (Fu et al., 2024) |
|  | - 1. Identity and stigma | “For example, recovery of lost personhood and meaningful occupation were important to participants: *Getting me life back. Yeah and get back in work and get back to the person I used to be*.” (Awenat et al., 2018) |
|  |  | “Other people found their suicide attempt difficult to deal with emotionally; they did not know what to say, or how to respond and it easier to ignore the person and stay away.” (Jackson et al., 2020) |
| 1. Feeling neglected by the system | - 1. The need for accessible, continuous and timely support after discharge | “They described being discharged a second time as *an awful experience* with the level of support that was provided when they were back in the community post-discharge.” (Bennewith et al., 2014) |
|  |  | “*I would prefer to have a 24 hour follow up because that is the critical part. You used to see all those people taking care of you and now you are isolated so, 24 hours would be better*” (Steinberg et al., 2024). |
|  | - 1. Desire for human care | “Interviewer: So when you were discharged, how did you feel? SU7: *Let down, that people didn’t really care. … It’s just like, “Go on home get on with your life.” And you think, well, what life*?” (Owen-Smith et al., 2014) |
|  |  | “Receiving support from someone genuine, who had time for them was highly valued: *Oh it was absolutely amazing, it was such an appreciated feeling…. and it felt like somebody was there for you, that you weren’t alone, neglected and just, abandoned, by everybody*” (Redding et al., 2017) |
| 1. Taking the reins on recovery | - 1. Autonomy and control | “Of no longer being an inpatient “recovering” from one’s suicidality (a temporary yet significant “pillar” of self and/or identity); and transitioning back to a metaphysical situation where the person has (little or) no option but to experience the “freedom” and responsibility to care for oneself (once again), despite not feeling up to the task.” (Cutcliffe et al., 2012a) |
|  |  | “…you are locked in, you don’t decide by yourself whether you go out, and you don’t decide by yourself whether to be discharged, and they can define what they want within a frame where it is not visible to many others” (Hagen et al., 2020) |
|  | - 1. Responsibility and self-development | “Just my own research, and that has that has been the biggest factor in my recovery has not come from somebody else it’s come from me.” (Brenisin et al., 2023) |
|  |  | “*External inevitability is beyond our power to change. I can only make internal changes, ... offering help and actively listening to others. Assisting others can cultivate a sense of warmth and connection between people*” (Fu et al., 2024) |
